# Structural and energetic analysis of metastable intermediate states in the E1P–E2P transition of Ca^2+^-ATPase

**DOI:** 10.1073/pnas.2105507118

**Published:** 2021-09-30

**Authors:** Chigusa Kobayashi, Yasuhiro Matsunaga, Jaewoon Jung, Yuji Sugita

**Affiliations:** ^a^Computational Biophysics Research Team, RIKEN Center for Computational Science, Kobe 650-0047, Japan;; ^b^Graduate School of Science and Engineering, Saitama University, Saitama 338-8570, Japan;; ^c^Theoretical Molecular Science Laboratory, RIKEN Cluster for Pioneering Research, Saitama 351-0198, Japan;; ^d^Laboratory for Biomolecular Function Simulation, RIKEN Center for Biosystems Dynamics Research, Kobe 650-0047, Japan

**Keywords:** calcium ion pump, protein–lipid interactions, molecular dynamics, conformational change, free energy analysis

## Abstract

Sarcoplasmic reticulum (SR) Ca^2+^-ATPase transports two Ca^2+^ ions from the cytoplasm to the SR lumen against a large concentration gradient. X-ray crystallography has revealed the atomic structures of the protein before and after the dissociation of Ca^2+^, while biochemical studies have suggested the existence of intermediate states in the transition between E1P⋅ADP⋅2Ca^2+^ and E2P. Here, we explore the pathway and free energy profile of the transition using atomistic molecular dynamics simulations with the mean-force string method and umbrella sampling. The simulations suggest that a series of structural changes accompany the ordered dissociation of ADP, the A-domain rotation, and the rearrangement of the transmembrane (TM) helices. The luminal gate then opens to release Ca^2+^ ions toward the SR lumen. Intermediate structures on the pathway are stabilized by transient sidechain interactions between the A- and P-domains. Lipid molecules between TM helices play a key role in the stabilization. Free energy profiles of the transition assuming different protonation states suggest rapid exchanges between Ca^2+^ ions and protons when the Ca^2+^ ions are released toward the SR lumen.

## Significance

Ion pumps (or P-type ATPases) are membrane proteins, which transport ions through biological membranes against a concentration gradient, a function essential for many biological processes, such as muscle contraction, neurotransmission, and metabolism. Molecular mechanisms underlying active ion transport by ion pumps have been investigated by biochemical experiments and high-resolution structure analyses. Here, the transition of sarcoplasmic reticulum Ca^2+^-ATPase upon dissociation of Ca^2+^ is investigated using atomistic molecular dynamics simulations. We find intermediate structures along the pathway are stabilized by transient interactions between A- and P-domains as well as lipid molecules in the transmembrane helices.

Sarcoplasmic reticulum Ca^2+^-ATPase (SR Ca^2+^-ATPase or SERCA1a) is a representative P-type ATPase that transports Ca^2+^ ions against a 10^4^ times concentration gradient across the SR membrane. The transport mechanism was originally described by E1/E2 theory, whereby the protein alternates between Ca^2+^ high-affinity E1 and low-affinity E2 states. A more-detailed reaction cycle requires multiple steps, including the binding/dissociation of Ca^2+^, H^+^-counter transport, ATP-binding and hydrolysis, phosphorylation/dephosphorylation of Asp351, and the dissociation of ADP and Pi (1–3) (*SI Appendix*, Fig. S1). Structurally, the Ca^2+^-ATPase consists of three cytoplasmic domains (actuator; A, nucleotide-binding; N, and phosphorylation; P) and 10 transmembrane (TM) helices (M1-M10) (4, 5). Two Ca^2+^-binding sites are located in M4-M6 and M8 (6), while a nucleotide, ATP or ADP, is bound at the N–P domain interface (7). Functional interconnections between the cytoplasmic domains and TM helices are necessary in the reaction cycle (8, 9).

**Fig. 1.**
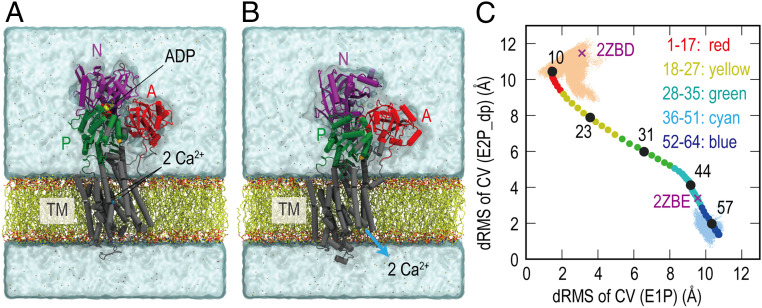
Structures of SR Ca^2+^-ATPase embedded in a DOPC membrane in E1P⋅ADP⋅2Ca^2+^ (PDB ID: 2ZBD) (*A*) and E2P (PDB ID: 2ZBE) (*B*). The three cytoplasmic domains (A, N, and P) are colored in red, purple, and green, respectively. (*C*) A structural transition pathway predicted using the mean-force string method is projected onto a two-dimensional map along with the two distance RMSDs (*dRMS*) from the representative structures in MD simulations of E1P and E2P_dp. In the simulation, Glu908 at the Ca^2+^-binding sites is protonated to mimic the same protonation states of E1P. Only the atomic coordinates that are involved in the collective variables (CVs) are used for *dRMS* calculations. The five substates (SSs) are defined along the pathway via the fixed radius clustering method (1 to 17: red; 18 to 27: yellow; 28 to 35: green; 36 to 51: cyan; and 52 to 64: blue). Among the 64 images, 5 images (10, 23, 31, 44, and 57) are selected as five representative SSs.

Molecular mechanisms underlying Ca^2+^ uptake by the ATPase have been investigated in biochemical experiments as well as structural studies. In particular, crystal structures of the Ca^2+^-ATPase, which represent different physiological states in the cycle (*SI Appendix*, Fig. S1), have provided essential information for understanding structure–function relationships (8, 9). Each crystal structure well explains the results of mutagenesis (3, 10), limited proteolysis studies (11, 12), and other biochemical experiments. Comparisons between multiple crystal structures provide direct evidence on how conformational changes of the Ca^2+^-ATPase take place from one step to another in the cycle. For instance, crystal structures that represent E1P⋅ADP⋅2Ca^2+^ and E2P (Fig. 1 *A* and *B*) reveal important conformational changes to release Ca^2+^ toward the SR lumen: 1) the A-domain rotates ∼90°; 2) the threonine–glycine–glutamate–serine (TGES) loop in the A-domain reaches the phosphorylated Asp351 in the P-domain (13–15); 3) M1-M6 are rearranged to open the luminal gate for the dissociation of Ca^2+^. Despite the increasing structural information, there are still unresolved questions. A series of biochemical studies suggested the existence of two intermediate states, E1P⋅2Ca^2+^ and E2P⋅2Ca^2+^. However, atomistic structures and their energetics in these intermediate states would be required to understand their functional roles.

There are several computational tools to predict conformational transition pathways of proteins, such as morphing (16), normal mode analysis (17), or molecular dynamics (MD) simulation based on coarse-grained or atomistic models (18–21). However, large conformational changes of the Ca^2+^-ATPase happen on the milliseconds or slower time scales, which are not accessible in brute-force MD simulations (18–21) even when using MD-specialized supercomputers, such as Anton/Anton 2 (22, 23) or MDGRAPE-4A (24). In this study, we perform atomistic MD simulations with an enhanced conformational sampling method to investigate the conformational pathway and free energy profile in the transition between E1P⋅ADP⋅2Ca^2+^ and E2P. We utilize the mean-force string method (25) for obtaining one of the most probable transition pathways. The free energy profile along the pathway is then calculated with umbrella sampling (26). The same approach has been previously applied to adenylate kinase in solution (27) and multidrug transporter AcrB (28) and the ABC heme transporter (29) in biological membranes. A similar method, the string method with swarm trajectories (30), has been applied to several membrane proteins (31, 32). Das et al. applied the method to four conformational transitions of the Ca^2+^-ATPase, including the same step examined in the current study (33).

In the current simulation study, we intend to compare the simulation results with those of existing structural and biochemical studies. In particular, the two intermediate states (E1P⋅2Ca^2+^ and E2P⋅2Ca^2+^) and the Ca^2+^-ATPase–lipid interactions in the E1P–E2P transition are examined using the simulation trajectories. The predicted interactions between phospholipids and basic sidechains of Ca^2+^-ATPase are compared with recent X-ray crystallography studies (34). We also investigate the effect of protonation states in the E1P–E2P transitions from atomistic MD simulations, which is difficult via experimental studies. By integrating structural, biochemical, and computational results, we shed light on the structural and energetic features of the E1P–E2P transition of the Ca^2+^-ATPase in unprecedented detail.

## Results

### MD Simulations of SR Ca^2+^-ATPase in the E1P⋅ADP⋅2Ca^2+^ and E2P States.

MD simulations of SR Ca^2+^-ATPase in E1P⋅ADP⋅2Ca^2+^ and E2P are performed to examine the atomic fluctuations (Fig. 1). Modeling and simulation methods are described in *Materials and Methods* and *SI Appendix*. We denote the simulation systems representing E1P⋅ADP⋅2Ca^2+^ as E1P, while E2P_dp and E2P denote two systems in E2P with different protonation states, respectively (*SI Appendix*, Table S1): E2P_dp has the same protonation state as E1P (only Glu908 is protonated), while Glu771, Asp800, and Glu908 are protonated in E2P. In the simulations of E1P, E2P_dp, and E2P, the Cα atoms’ RMSDs from the starting structures increase above 4 Å (*SI Appendix*, Fig. S2). Since each cytoplasmic domain and the TM helices are individually stable (RMSD < 3 Å), the increase in RMSD of the whole structure suggests large domain motions. A hierarchal clustering provides a representative structure at the cluster center of each MD simulation trajectory (*SI Appendix*, Fig. S3). Large deviations are observed only in the N-domain between the three MD simulations when representative snapshots are superimposed on the starting crystal structures. Interestingly, large motions in M4 and M6 are observed in the simulation of E2P_dp, probably due to mismatches between the TM structure and protonation states of the Ca^2+^-binding residues. The luminal gate in E2P_dp is more open than in the crystal structure, which may reflect the structural changes occurring just after the dissociation of Ca^2+^.

### The Structure Transition from E1P⋅ADP⋅2Ca^2+^ to E2P.

We next examine how structure changes happen from E1P⋅ADP⋅2Ca^2+^ to E2P in the three simulations. First, we use the E1P system and perform MD simulations with the mean-force string method for predicting one of the most probable transition pathways between E1P⋅ADP⋅2Ca^2+^ and E2P. Due to strong Ca^2+^–protein interactions, we do not expect that the two Ca^2+^ ions bound at the TM binding sites are fully released toward the SR lumen, even in the images close to E2P. After the simulation of the first model (E1P), we extract a protein structure from image 50. We then carry out the second simulation with the string method between this image and the representative structure of E2_dp, assuming the same protonation state as E2_dp. In the third simulation of the string method, we connect image 50 and the representative structure of E2P simulation, using the E2P protonation state (*SI Appendix*, Fig. S3). In this way, we will answer several questions regarding what series of conformational changes occur in the cytoplasmic and TM domains, how the motions of the cytoplasmic domains are coupled with those of TM helices, and how the exchange between Ca^2+^ and protons occurs at the TM binding sites. Technical details of the mean-force string method are described in *Materials and Methods* and *SI Appendix*.

**Fig. 2.**
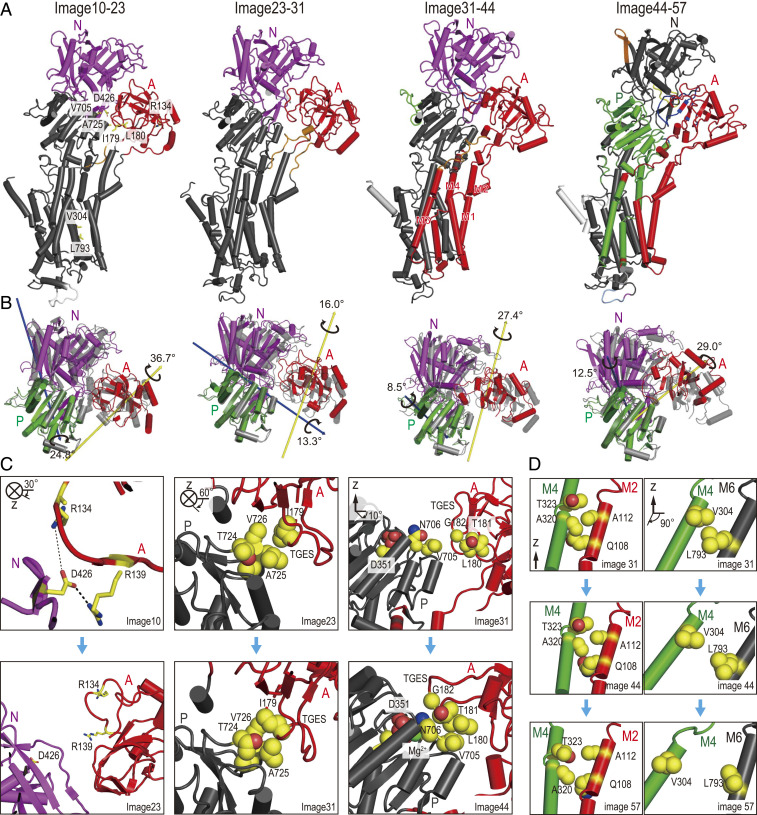
Conformational changes of the Ca^2+^-ATPase in the E1P–E2P transition. (*A*) MT for centroid images of SSs on the pathway from E1P to E2P_dp. From *Left* to *Right*, four representative SS transitions between images 10 to 23, 23 to 31, 31 to 44, and 44 to 57. Rigid domains derived by MT are shown with different colors at each SS transition. In other words, parts with the same color are considered as a single rigid domain. The domains are further divided (as shown in white, blue, orange, yellow, and green parts), and the patterns of division become more complex at latter transitions (31–44 and 44–57). Selected residues are shown in stick representation with labeling on the SS transition 10 to 23 (*Left*). (*B*) Movements of the cytoplasmic domains between central images of two adjacent SSs. Colored and gray structures represent later and earlier SS structures, respectively. A-, P-, N-domains are shown in red, green, and magenta. Yellow and blue arrows indicate the rotation axes of the A-, and N-domains, respectively, with respect to the P-domain. (*C*) Changes of contacts between the cytoplasmic domains. *Left*, *Middle*, and *Right* correspond to the SS transitions between images 10 to 23, 23 to 31, and 31 to 44, respectively. Protein colors are rigid domains at each SS transition in (*A*). (*D*) Conformational changes of M2, M4, and M6. *Left* and *Right* represent the hydrophobic core in the cytoplasmic part of M2 and M4 and the luminal gate between M4 and M6 in images 31, 44, and 57 (from *Top* to *Bottom*). M2, M4, and M6 are shown in red, green, and black.

The initial paths of the three string simulations are derived from the targeted MD (TMD) method (*SI Appendix*, Table S2) and are then optimized toward one of the most probable ones by the mean-force string method (*SI Appendix*, Table S3 and Fig. S4). The optimized pathway in the first simulation is represented by the 64 images on a two-dimensional map along with the distance RMSDs from representative MD structures of E1P and E2P, respectively. The 64 images are divided into five substates (SSs) using the fixed-radius clustering in Multiscale Modeling Tools for Structural Biology (MMTSB) (35, 36) (Fig. 1*C*). The clustering results do not change significantly with a different radius. The centroid structures in the five SSs are selected from images 10, 23, 31, 44, and 57. The structures between adjacent SSs are compared using Motion Tree (MT) (37), which identifies rigid structural units based on the hierarchical clustering of local structures. In Fig. 2*A*, four SS transitions are described via moving rigid structural units highlighted in different colors. In the first two SS transitions, the N- and A-domains move independently. MT detects the A-domain and M1–M4 as a single moving domain in the third SS transition (between images 31 and 44), suggesting that the coupling of the A-domain rotation with the TM helices starts from the metastable intermediate. Finally, M1–M2 and M3–M4 move as independent rigid structural units, suggesting the gating motions for releasing Ca^2+^ ions toward the SR lumen. In this step, the A-domain rotation is coupled with M1–M2, and the P-domain inclination takes place together with M3–M4 (in green, Fig. 2 *A*, *Right*).

### The Relationship between the Cytoplasmic Domain Motion and the Interdomain Interaction.

To understand the cytoplasmic domain motion, we compute the rotational axes and angles of the A- and N-domains relative to the P-domain using the domain selection (DS) method (38) (Fig. 2*B* and *SI Appendix*, Fig. S5). In the first SS transition, both the A- and N-domains show large rotational motions (37° and 25°). However, their rotational axes are almost perpendicular to each other, suggesting their independent motions. In the second and third SS transitions, the rotational axis of the A-domain is almost the same and is parallel to the membrane normal (the *z*-axis). The TGES loop of the A-domain now approaches the phosphorylated Asp351 in the P-domain. In the final step, from image 44 to 57, DS detects a large motion of the A-domain relative to the P-domain. This large rotation is possible due to the P-domain inclining and pushing M4 toward the SR luminal side.

These rotational motions start just after the disconnection of ADP from the phosphorylated Asp351 (*SI Appendix*, Fig. S6). In the E1P⋅ADP⋅2Ca^2+^ crystal structure, the A- and N-domains are interconnected via salt bridge interactions between Arg134/Arg139 in the A-domain and Asp426 in the N-domain (Fig. 2 *C*, *Top Left*). Biochemical experiments suggested that the salt bridge interactions play an essential role in phosphoenzyme formation (39). A repulsion between the phosphate groups in ADP and the phosphorylated Asp351 may trigger the conformational changes at the interface of the A- and N-domains. (*SI Appendix*, Figs. S6 and S7). Breaking of the salt-bridge interactions allows independent motions of the A- and N-domains in the first SS transition. When the TGES loop approaches the phosphorylation site in the P-domain during the second SS transition, hydrophobic interactions between Ile179 in the A-domain and Ala725/Val726 in the P-domain seem to be important (Fig. 2 *C*, *Middle*). In the next step, different hydrophobic interactions between Leu180 in the A-domain and Val705 in the P-domain are formed. The clockwise rotation of the A-domain seems to be precisely controlled using such a hydrophobic rail on the P-domain. Importantly, the distance between the TGES loop and the phosphorylated Asp351 decreases almost linearly (*SI Appendix*, Fig. S7 *B*, *Bottom*) during the SS transitions. The transient hydrophobic interactions between the A- and P-domains can guide functionally important amino acid residues to meet almost at the end of the transition.

### Rearrangement of the TM Helices during the Reaction.

To reduce the Ca^2+^-binding affinity in E2P, rearrangement of M1–M6 is necessary. In particular, a downward shift of M4 and a rotational motion of M6 are known as the two key conformational changes to open the luminal gate (40, 41). The exact sequence of the M1–M6 rearrangement is examined in terms of three structural properties in the optimized images of the first string-method calculation: the angle defined with the Cα atoms of Val89, Gly105, and Pro124 in M2 (M2 bending angle, *SI Appendix*, Fig. S8 *A*, *Top*), the distance between the cytoplasmic parts of M2 and M4 (M2–M4 distance, *SI Appendix*, Fig. S8 *A*, *Middle*), and the distance between the sidechain atoms of Val304 in M4 and Leu793 in M6 (M4–M6 distance, *SI Appendix*, Fig. S8 *A*, *Bottom*). In the first two SSs (images 10 and 23), these values remain close to those in the E1P⋅ADP⋅2Ca^2+^ crystal structure. The M2 bending angle decreases in the second SS transition because the rotation of the A-domain pulls the A–M2 linker. The M2 bending angle is maintained in the later transitions, which allows the distance between the cytoplasmic halves of M2 and M4 to enlarge slightly. The region is stabilized via hydrophobic interactions observed in the E1P crystal structure (Fig. 2 *D*, *Middle Left*). The reduced interaction in the cytoplasmic halves of M2 and M4 seems important for the downward shift of M4. The M4–M6 distance is drastically changed in the last SS transition (from image 44 to 57), and the hydrophobic core formed by Val304 in M4 and Leu793 in M6 is totally broken (Fig. 2 *D*, *Bottom Right*). MT indicates that the M1–M2/A-domain (red) and M3–M4/P-domain (green) move together as rigid moving domains (Fig. 2 *A*, *Right*). The rotational motion of M6 is likely independent of the cytoplasmic domain motions and happens due to mismatched hydrophobic and electrostatic interactions after the M1–M4 motions.

The sequence of events and the coupling between the cytoplasmic and TM domain motions substantially agree with inferences from structural studies and biochemical experiments. In particular, the rate of phosphoenzyme isomerization from E1P to E2P is affected by the mutations in the cytoplasmic part of M2 and M4 (42, 43). Both this simulation and the existing experiments point to the importance of the hydrophobic interactions in the TM regions. Large motions of M4 and M6 are only possible once the interactions are reduced. MT, DS, and analysis of key sidechain interactions in the four SS transitions could integrate our understanding of the coupling of the cytoplasmic domain motions with the rearrangements of M1–M4. This is useful for shedding light on the molecular mechanisms of how the luminal gate is opened.

### Interactions between Lipid Molecules and TM Helices.

**Fig. 3.**
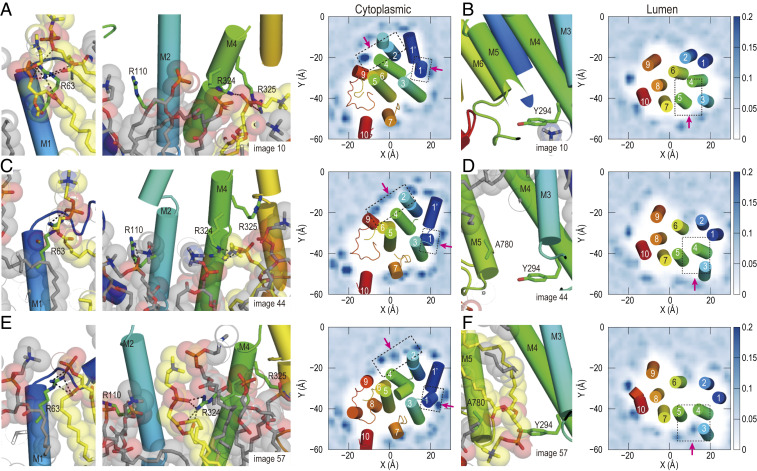
Interactions between the TM helices and lipid molecules in the cytoplasmic (*A*, *C*, and *E*) and lumen (*B*, *D*, and *F*) sides. (*Left*) Snapshot of M1–M6 helices and surrounding lipid molecules. (*Right*) Distribution of phosphorus atoms in lipid molecules around the protein. Dotted boxes mark the areas of the snapshots shown on the *Left*, and pink arrows indicate the direction of view. C atoms of the lipid molecules bound to the ATPase are shown in yellow, and C atoms of the other lipid molecules are shown in gray. The protein’s backbone is shown in rainbow spectrum with the N terminal in blue. C, N, and O atoms in the sidechain are shown in green, blue, and red, respectively.

The rearrangements of M1–M4 helices can also be affected by protein–lipid interactions. Recent crystallographic studies visualize annular lipid molecules in four prominent physiological states [E1⋅2Ca^2+^, E1⋅AlF_4_^−^⋅ADP⋅2Ca^2+^, E2⋅AlF_4_^−^ (TG), and E2(TG)] (34). Two key questions in the current analysis are whether atomistic MD simulations reveal the corresponding protein–lipid interactions and how protein–lipid interactions change during the four SS transitions. In Fig. 3, the distribution of phosphorus atoms of the lipid molecules surrounding the protein is calculated using images 10, 44, and 57. Recall that MT has suggested that the rearrangements of M1–M4 start from the third SS transition. In fact, we could observe characteristic protein–lipid interactions in each SS both on the cytoplasmic and lumen sides of the lipid bilayer.

In image 10, Arg110 in M2 and Arg324 and Arg325 in M4 associate with the head groups of lipid molecules on the cytoplasmic side (Fig. 3*A*), while no strongly bound lipid molecules are observed on the lumen side. This is reasonable because the TM helices on the luminal side are packed tightly to maintain a closed luminal gate before the last SS transition. This observation agrees with the crystallographic studies (34) and biochemical experiments (42). In Fig. 3 *C* and *E*, we observe that Arg63 and Arg324 interact with dioleoyl phosphatidylcholine (DOPC) lipids, and these are the same interactions seen in the E1P⋅ADP⋅2Ca^2+^ crystal structure. After disruption of the hydrophobic core formed by the cytoplasmic halves of M2 and M4, a lipid molecule fills the space between the two TM helices (Fig. 3*E*). After the luminal gate opens, another lipid molecule is inserted in the space between M3, M4, and M5 on the lumen side (Fig. 3*F*). The crystallographic study does not show a lipid filling the lumen space in two end points of E1P⋅ADP⋅2Ca^2^ and E2P. Our simulation study shows the importance of the protein–lipid interactions in the intermediate structures just after the luminal gate opens.

### Free Energy Profiles along the Reaction Pathways for Different Protonation States.

**Fig. 4.**
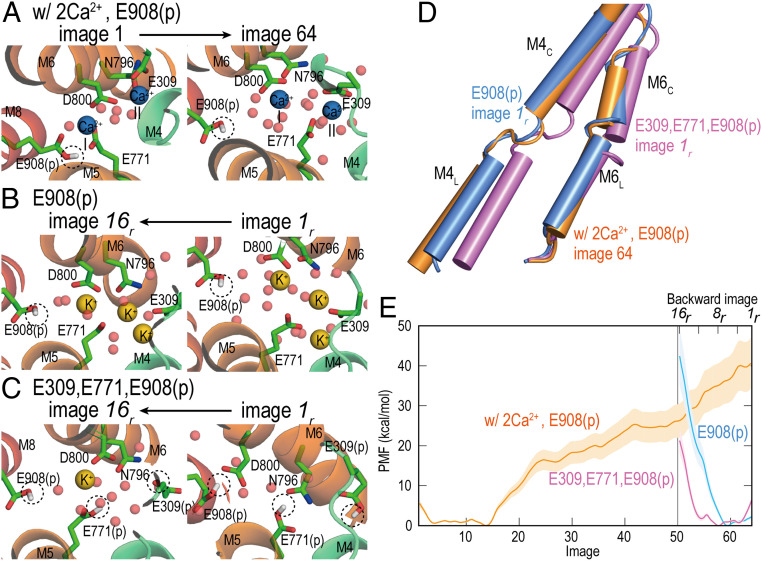
Energetics of the E1P–E2P transitions. The structures near the Ca^2+^-binding sites in the first and last images in the string-method simulations (*A*) from E1P to E2P_dp, (*B*) from E2P_dp to image 50 (image *16_r_*), and (*C*) from E2P to image 50 (image *16_r_*). The protonated Glu are highlighted with dashed circles. Ca^2+^, K^+^, and water are shown in blue, yellow, and red spheres. Only sidechains of the Ca^2+^-binding residues are shown with stick representations. C, N, and O atoms in the stick representations are shown in green, blue, and red, respectively. The protein’s backbone is shown in rainbow spectrum with the N terminal in blue. (*D*) The structures of M4 and M6 in images 64 from E1P to E2P_dp (orange), *1_r_* from E2P_dp to image 50 (blue), and *1_r_* from E2P to image 50 (pink). (*E*) Free energy profiles obtained in the string-method calculations using E1P (orange), E2P_dp (blue), and E2P (pink). In E1P and E2P_dp, only Glu908 is protonated, while Glu309, Glu771, and Glu908 are protonated in E2P.

We investigate protein–Ca^2+^ interactions despite the limited accuracy of the current force field model of divalent cations. There are two Ca^2+^-binding sites in the TM domain: In site-I, Ca^2+^ is bound to the sidechains of Glu771, Asp800, Glu908, while the site-II Ca^2+^ is stabilized with the sidechain atoms of Glu309, Asn796, Asp800, and backbone carbonyl atoms in M4 (Fig. 4*A*). In image 64 of the first string-method simulation, many water molecules enter because of the opening of the luminal gate. Although both Ca^2+^-binding sites are greatly disrupted, two Ca^2+^ ions stay nearby. The site-I Ca^2+^ keeps the interaction with the sidechain of Asp800, and the site-II Ca^2+^ is bound with Glu771 and Glu309. These interactions may happen just after the rearrangements of M1–M4. In the second and third string-method calculations, the luminal gate is wide open in image 50 due to the rearrangement of M1–M6 (*SI Appendix*, Figs. S9–S11). In the second simulation (E2P_dp), not only water molecules but also K^+^ ions penetrate from the lumen side. Both images *1*_*r*_ and *16*_*r*_ (same as image 50 in the first simulation except for Ca^2+^ ions) contain three K^+^ ions at the binding sites (Fig. 4*B*). Interestingly, only one K^+^ is observed at the binding sites in image *16*_*r*_ of the third simulation, and no K^+^ exists in image *1*_*r*_ when Glu309, Glu771, and Glu908 are protonated (Fig. 4*C*). In image *1*_*r*_, the luminal gate is rather closed compared to that in the second simulation (E2P_dp) (Fig. 4*D*). These results suggest that rapid exchanges between Ca^2+^ and protons are necessary under physiological conditions just after releasing Ca^2+^ ions toward the SR lumen for timely closure of the luminal gate and prevent the uptake of Ca^2+^ to the binding site.

Using umbrella sampling, we compute the potential of mean forces (PMFs) from the three simulations. The PMF obtained in the first simulation shows a flat curve between images 1 and 14 (Fig. 4*E*). Then, the curve rises smoothly until image 50. Due to the strong protein–Ca^2+^ interactions, we do not observe the second minima toward image 64 (E2P). The second rise in PMF results from a mismatch between the TM structures and the Ca^2+^-binding states. The barrier height in the third simulation, where the same protonation state of the E2P crystal structure exists, is greatly reduced compared to that in the second simulation with the E1P protonation state. This also supports the existence of a quick exchange of Ca^2+^ and protons at the ion-binding sites. If we integrate the three PMF curves, the complete energetics of the transition from E1P⋅ADP⋅2Ca^2+^ to E2P is described.

## Discussion

### Molecular Mechanism of the E1P–E2P Transition in SR Ca^2+^-ATPase.

**Fig. 5.**
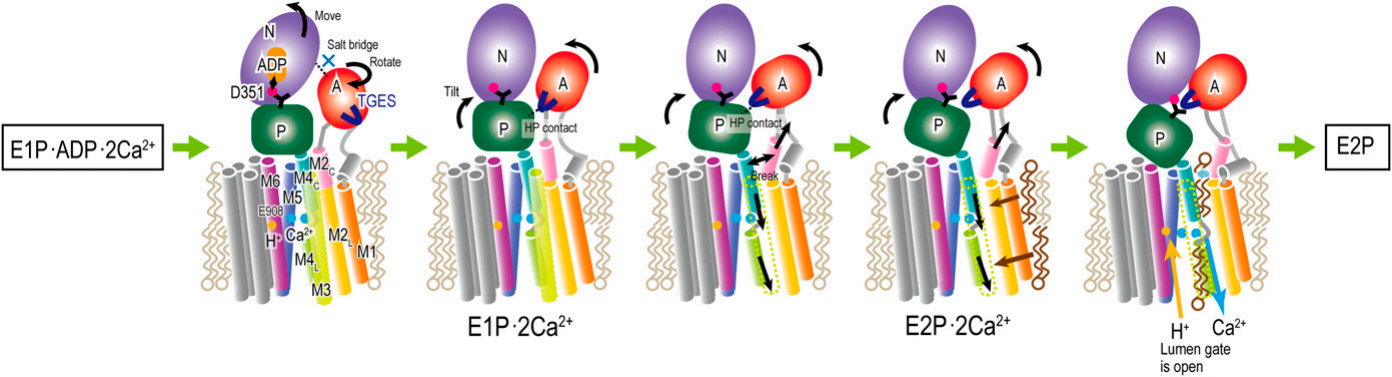
A schematic view of the transition from E1P⋅ADP⋅2Ca^2+^ to E2P. Structural features of the two intermediates (E1P⋅2Ca^2+^ and E2P⋅2Ca^2+^) are predicted in the current simulations. HP contact indicates the transient hydrophobic interactions between A- and P-domains.

In this study, we examine structures and energetics of the E1P–E2P transitions in SR Ca^2+^-ATPase using atomistic MD simulations. The key results are summarized in Fig. 5. In the starting structure of the transition, two Ca^2+^ ions are stably bound at the TM binding sites, and ADP/Mg^2+^ is bound at the cytoplasmic binding sites formed with the N- and P-domains. The crystal structure in the E1P⋅ADP⋅2Ca^2+^ state shows a compact cytoplasmic structure, which is stabilized by ADP (between the N- and P-domains) and sidechain salt bridge interactions between the N- and A-domains. The disconnection of ADP from the phosphorylated Asp351 and break-up of the salt bridges happen spontaneously without large energetic costs, allowing the A-domain to rotate independently in the beginning. The TGES loop in the A-domain needs to reach the phosphorylated Asp351 in the P-domain at the end of the transition. The large arc-like movement of the A-domain is guided not only by sidechain interactions observed in the crystal structures but also by transient interactions along the way. Hydrophobic interactions with the P-domain act as a rail for fixing the rotation axis of the A-domain. The coupling of the A-domain rotation with the rearrangement of M1–M6 helices occur in almost the final stage of the E1P–E2P transition. One of the key events to initiate the coupling is the change in hydrophobic interactions at the cytoplasmic halves of M2 and M4 helices. With the interactions severed, it is easier for the A-domain rotation and for the inclination of the P-domain to shift M4 downward. The subsequent rotation of M6, which is necessary to open the luminal gate for releasing Ca^2+^ ions, seems to be driven by mismatched sidechain interactions following the M1–M4 movements.

Energetically, the E1P–E2P transition starts with a flat landscape during which ADP is released, which causes the N- and P-domains to move independently. The rotation of the A-domain causes an uphill energy landscape, suggesting that the intermediate structures are not so stable compared to E1P and E2P. In almost the final stage, the Ca^2+^ ions are released toward the lumen, and protonation of the Ca^2+^ binding residues happens quickly to keep the structural integrity of the mobile TM helices. The opening of the luminal gate means a reduction of protein sidechain interactions, and in compensation, lipid molecules play an important role in stabilizing TM helices. The phosphate head group of a lipid molecule transiently pairs with basic residues in TM region, while the hydrophobic acyl chains fill cavities between TM helices. The current MD simulation studies add information missing from experiments and contribute to a better understanding of the E1P–E2P transition. In addition to the decrease of the interactions between Ca^2+^ and the binding residues, entropy would be another factor for the energetics in the release of Ca^2+^. As shown by Rubí et al. (44), the pore geometry formed by the opening of the luminal gate increases the entropy of the ion toward the lumen and would facilitate the release. Overall, both the enthalpy and the entropy can contribute to the rapid Ca^2+^ release from the transient E2P_dp state.

### Relations with Previous Experimental/Computational Studies of SR Ca^2+^-ATPase and Other P-Type ATPases.

Here, we discuss relationships between this work and previous experimental/computational studies of SR Ca^2+^-ATPase and other P-type ATPases. The structure changes during the E1P–E2P transitions that we have observed from the simulations basically agree with the model of the conformational transitions deduced from the crystal structures representing their two end points. The structure changes we have predicted are in good agreement with the previous study by Das et al. (33). Although different schemes and software were used in the two string-method simulations, the consistent results could testify to the reliability of the computational studies. The protein–lipid interactions predicted in the simulations are consistent with the experimental visualization of the surrounding lipid molecules (34). The accord strengthens the case for a physiologically meaningful role of the protein–lipid interactions in the stability and function of the ATPase. Suzuki and coworkers showed that the rotation of the A-domain and conformational changes of the A–M linkers play an important role in the E1P–E2P transition (42, 43, 45, 46). Their works pointed to the existence of two metastable intermediate states, E1P⋅2Ca^2+^ and E2P⋅2Ca^2+^, along the transition (11, 12, 45, 47). In those states, they consider that the locations of the A-domain and, as a result, the A–M linker structures of these two metastable states, are different from the crystal structure of E1P·ADP⋅2Ca^2+^. In the current study, the second and fourth SSs may correspond to the E1P⋅2Ca^2+^ and E2P⋅2Ca^2+^ metastable structures, respectively. Single-molecule Förster resonance energy transfer experiments of bacterium Ca^2+^-ATPase also detected a metastable intermediate state like E2P⋅2Ca^2+^ in the E1P–E2P transition (48). They also showed that the formation of a hydrophobic cluster around Tyr122 in the cytoplasmic part of M2 is shown in the opening luminal gate (46). However, this hydrophobic cluster is not observed in our simulations.

There are several other studies which have investigated the possibility of metastable intermediates in the E1P–E2P transition of different P-type ATPases, such as Na^+^,K^+^-ATPase (49–52) and *Listeria* monocytogenes Ca^2+^-ATPase (48). Although P-type ATPases share a common structural architecture and reaction cycles, they have diverse functions in biological membranes. Elucidating the intermediate structures between crystal structures in different physiological states might be a key for better understanding of each function. In the recent X-ray study of Na^+^,K^+^-ATPase referred to, structures of multiple metastable intermediates of E2P bounds with a single Mg^2+^ and/or a single K^+^ ion are described. In addition, the protein binds a cardiotonic steroid molecule in the cavity of extracellular side of M4–M6 (52). The steroids are specific inhibitors of the pump. To understand the biological functions of these ATPases in more detail, the structural and energetic analysis of intermediate structures should be meaningful.

### Limitations of the Current Simulation Methods and Future Perspectives.

Here, we discuss the limitation of the current simulation models and methods. As a whole, the current simulation studies provide qualitatively meaningful results to add missing information of the E1P–E2P transitions of Ca^2+^-ATPase. However, if we aim to compare the simulation results with experimental measurements quantitatively, there is room in models and methods to be improved. As we discussed already, the problem related to divalent cation models in the classical force fields has been often discussed. In the current study, due to these problems, the PMF in Fig. 4*E* seems to be an overestimation, and there is a mismatch between Ca^2+^–protein interaction and protein structures near the final stages of the transition. Because of this, the free energy curve is uphill right up until E2P. A new Ca^2+^ model that reduces the strong interactions here may be useful. Recently, an updated Ca^2+^ model successfully simulated the translocation of Ca^2+^ through the ryanodine receptor (53).

Application of the constant-pH method increases the power of molecular simulations where the protonation states change in different states of a protein (54, 55). There are several successful applications of the constant-pH method to membrane channels and transporters (56). However, it is computationally expensive if the method is coupled with multicopy enhanced sampling methods like the mean-force string method simulations. The enhanced sampling method itself requires a number of images (or replicas). If more replicas are added in the constant-pH calculations, the total number of replicas might not be able to handle, even using modern supercomputers. Our current sampling scheme, a combination of the mean-force string method and umbrella sampling, may be criticized on several issues. The string method provides the local optimization of a reaction pathway, and the final pathway may not be an overall free energy global minimum. Also, due to the intrinsic flexibilities of biomolecules, more than one pathway may exist. Nevertheless, in this study, we provide a detailed structural analysis of intermediate states by MT (37), DS (38), and others, the energetic analysis with different protonation states, and analysis of protein–lipid interactions that stabilize TM structures. These should be sufficiently meaningful to answer unresolved questions on the E1P–E2P transition in the Ca^2+^-ATPase.

## Materials and Methods

### MD Simulation.

Simulation systems in E1P⋅ADP⋅2Ca^2+^ and E2P of SR Ca^2+^-ATPase were constructed from the two crystal structures (Protein Data Bank [PDB] IDs: 2ZBD and 2ZBE). The protein was embedded in a DOPC lipid bilayer. According to a previous simulation study of E1⋅2Ca^2+^ (57), Glu908 is protonated in E1P⋅ADP⋅2Ca^2+^ (denoted E1P). In the E2P state, two simulation systems were constructed: one of which has the same protonation state as E1P⋅ADP⋅2Ca^2+^ (denoted E2P_dp), and the other with a different protonation state, in which Glu309, Glu771, and Glu908 are protonated as in the E2P crystal structure (denoted E2P). Detailed simulation methods are described in *SI Appendix*, and the notations and configurations are summarized in *SI Appendix*, Table S1.

### Simulations for Detecting the Pathway from the E1P State to the E2P States.

The pathway from E1P to E2P_dp was calculated as follows. An initial pathway consisting of 64 images was generated by a 200-ns TMD simulation. Then, a string-method simulation was performed, first for 40 ns without updating the images and next for 60 ns while updating the images. To calculate free energy profiles, umbrella sampling was applied to the obtained pathway. The last 46 ns from 50-ns simulation was used for the analysis. In addition, two pathways, with or without Ca^2+^ and in different protonation states of the Ca^2+^-binding residues were calculated. The first was applied from E2P_dp to the structure with closed luminal gate (image 50) and showed the opening/closing of the luminal gate without Ca^2+^. The second one simulates the pathway from E2P to image 50, which may mimic the structure after the binding of countertransport protons to the Ca^2+^-binding sites. Additional details are described in *SI Appendix*.

## Supplementary Material

Supplementary File

Supplementary File

## Data Availability

Input files and parameter set data have been deposited in GitHub at https://github.com/RikenSugitaLab/SERCA-E1P-E2P-pathway (58) (tag: v1.1).
