# Supplementary material for: Structural and energetic analysis of metastable intermediate states in the E1P–E2P transition of Ca2+-ATPase
Source: Proc Natl Acad Sci U S A. 2021 Sep 30;118(40):e2105507118. doi: 10.1073/pnas.2105507118 (PMC8501872; doi:10.1073/pnas.2105507118)
Supplement: Supplementary File [file pnas.2105507118.sapp.pdf]

PNAS
www.pnas.org

Supplementary Information for

Structural and energetic analysis of metastable intermediate states in the E1P-E2P transition of Ca^{2+}-ATPase

Chigusa Kobayashi,[1] Yasuhiro Matsunaga,[2] Jaewoon Jung,[1,3] Yuji Sugita[1,3,4 *]

[1]Computational Biophysics Research Team, RIKEN Center for Computational Science, 7-1-26 Minatojima-minamachi, Chuo-ku, Kobe 650-0047, Japan

[2]Graduate School of Science and Engineering, Saitama University, 255 Shimo-Okubo, Sakura ku, Saitama, Saitama 338-8570, Japan

[3]Theoretical Molecular Science Laboratory, RIKEN Cluster for Pioneering Research, 2-1, Hirosawa, Wako, Saitama 351-0198, Japan

[4]Laboratory for Biomolecular Function Simulation, RIKEN Center for Biosystems Dynamics Research, 6-7-1 Minatojima-minamimachi, Chuo-ku, Kobe, Hyogo 650-0047, Japan

* Corresponding to: Yuji Sugita

Email: sugita@riken.jp

This PDF file includes:

Supplementary text

Figures S1 to S13

Tables S1 to S3

SI References

Supplementary Information Text

Setup of simulation systems

Crystal structures of Ca^{2+}-ATPase in the E1P·ADP·2Ca^{2+} (2ZBD(1)) and E2P (2ZBE(2)) states were taken from the Protein Data Bank (PDB). AlF_4^- in 2ZBD and BeF_3^- in 2ZBE were replaced with a phosphate covalently bound to Asp351 (phosphorylated Asp351). K^+ bound at the P-domain plays a significant role in the dissociation of Ca^{2+}(3) so that it was inserted into the cavity of the P-domain. The Ca^{2+}-ATPase was embedded in a dioleoyl phosphatidylcholine (DOPC) lipid bilayer and the orientation of the protein with respect to the bilayer normal was determined by the PPM server.(4) There are four acidic residues (Glu309, Glu771, Asp800, and Glu908) at the Ca^{2+}-binding sites. Conforming to previous simulation results of E1·2Ca^{2+} (5), Glu908 was protonated in E1P·ADP·2Ca^{2+} (denoted E1P). In E2P, two simulation systems were constructed, one with the same protonation state as E1P·ADP·2Ca^{2+} (i.e., deprotonated E2P state, denoted E2P_dp), and the other with a different protonation state, in which Glu309, Glu771, and Glu908 were protonated (denoted E2P). The protonation states were determined in previous studies (5, 6), and PROPKA (7). Biological experimental results also showed that the molar ratio of Ca^{2+}/H^+ counter transport is 1 when pH is near neutrality (8, 9). Cavities inside the protein were filled with water molecules using DOWSER (10). The system was solvated and neutralized with 150 mM KCl. Simulation systems contained roughly 297,000 atoms. Table S1 summarizes the initial structures used in the MD simulations.

Conventional MD simulations

Conventional MD simulations of SR Ca^{2+}-ATPase in E1P, E2P_dp, and E2P were performed using the GENESIS MD program (11-13). The CHARMM 36 force field (14, 15) was used for the protein and lipid molecules. The modified TIP3P water model (16) was used. Parameters of ADP were taken from a previous work (17). Lennard-Jones (LJ) interactions of Ca^{2+} and Mg^{2+} were the same as those developed in a previous work (18). Constraints were assigned to all bonds involving hydrogen atoms using SHAKE or RATTLE (19, 20) and water molecules were kept rigid using SETTLE (21). Long-range electrostatic interactions were calculated using the particle-mesh Ewald summation method (22, 23). LJ interactions were smoothed over a 10-12 Å range with a force-based switching function (24). Multiple time step integration was used for calculating long-range electrostatic interactions (25). The time step of the MD integration was 2.5 fs. Long range interactions were evaluated every 2 steps, as previously tested. (26) Temperature (310.15 K) and pressure (1 atm) were controlled by the stochastic rescaling thermostat combined with the MTK barostat proposed by Bussi *et al* (27, 28). To keep interactions between the protein and ADP, a phosphate of phosphorylated Asp351, Mg^{2+}, Ca^{2+}, and K^+, restraint functions were applied during the simulations with a force constant of 10 kcal/mol/Å^2. Table S2 summarizes the simulation lengths of three MD and targeted MD (TMD) simulations. Representative structures of the three MD simulations were obtained from one of two top cluster centers of the trajectories by hierarchal clustering using the MMTSB tool. (29, 30) Root mean square displacements (RMSD) between the representative MD structures and the crystal structures were computed using the Cα atoms of Ca^{2+}-ATPase (Table S3).

One of the most probable transition pathways from E1P to E2P

Here, we used the mean-force string method (31) to determine one of the most probable pathways and umbrella sampling (32) to calculate the free energy profile along the pathway. The initial pathways in the string method simulations were obtained from short TMD simulations (33) (Table S2). Table S3 summarizes the MD simulation lengths in the string method and the umbrella sampling. Since classical force fields cannot treat proton transfer events explicitly, the whole pathways along the transition were calculated in the following two steps. First, a transition pathway from E1P to E2P_dp was obtained, while keeping two Ca^{2+} ions at the TM binding sites. Next, we simulated a reverse reaction without Ca^{2+} ions from E2P to an intermediate state (image 50)

obtained in the first step. In the second step, we performed two simulations, each of which assumes a different protonation state of the Ca^{2+}-binding residues, E2P_dp or E2P.

Most of the MD simulation conditions in the mean-force string-method and umbrella sampling~~,~~ were identical to those in conventional MD. Only the integrator and time step of the integration were different due to their availability in GENESIS software: Velocity-Verlet integrator with a 2-fs time step was used in the string method and umbrella sampling, while the multiple-time step integrator with 2.5 fs time step was employed in the conventional MD and TMD.

String method for calculating the reaction pathway

Initial pathways of the string method were obtained using TMD, which uses holonomic constraints on all heavy atoms of the protein (33). Initial images of the subsequent string method simulations were extracted using the ‘rpath_generator’ tool in GENESIS (13).

Collective variables (CVs) in the string method were chosen with special care to simulate domain motions smoothly. Cartesian coordinates of the Cα atoms in all residues were used to define CVs. In addition, the carbon atoms of selected sidechain atoms were added to CVs based on previous domain-motion analysis (28), several trials of TMD, and information of the experimental study (2). The selected sidechain atoms were 139 atoms from the residues Ile54, Leu61, Leu65, Leu66, Ala68, Glu90, Ile94, Gln177, Ser178, Leu180, Thr181, Gly182, Glu183, Ser184, Phe256, Trp272, Phe296, Lys297, Val304, Ala305, Ile307, Glu309, Asp351, Lys352, Thr353, Asn359, Glu439, Glu442, Arg489, Arg560, Thr625, Gly626, Asp627, Arg678, Asp703, Asn768, Glu771, Ile775, Pro789, Leu792, Leu793, Asn796, Thr799, Asp800, and Glu908. In total, the CVs contained 1133 atoms. (i.e. 3399 Cartesian coordinates, *SI Appendix*, Fig. S12)

In the string method calculations, the equilibration runs were performed without updating the images. To avoid extreme motions caused by a large force constant, the force constant of positional harmonic restraint was taken as 0.01 kcal/mol/Å^2 in the first 1 ns, and 0.1 kcal/mol/Å^2 in later steps. During the production runs, images were updated based on the calculated mean forces. The distance between neighboring images was kept equal. The mean forces were evaluated every 2 ps. The two end-images were fixed during the first 2 ns but were allowed to move in later steps. Tangential components of the pathway were eliminated from the mean force during updates of the terminal images. The RMSD from the initial images and the sum of distances of neighbor images during the simulation were monitored to check the convergence of the pathway. To superimpose simulation snapshots, the Cα atoms in the M5-M10 helices (referred to as the M5$_L$-M10 domain in our previous study (34)) were used and allowed to move only in the XY-plane.

Umbrella sampling simulations along the pathway

Umbrella sampling was performed by applying positional harmonic restraints with respect to the last images obtained in the string method calculations. The CVs were identical to those in the string-method calculations. Initial guesses of force constants were calculated from the length of the pathway and the number of images according to the method proposed by Park *et al*. (35). The final force constants were determined by trying several candidates around the initial guesses and adjusting them. The final values are shown in Table S3. The first 4 ns of the trajectories were not included for potential of mean force (PMF) calculations and the analysis for conformational changes. *SI Appendix*, Fig. S13 shows that the trajectories cover the reaction pathways. The MBAR analysis (36, 37) was applied to obtain weights for the restraint-free coordinates from the trajectory with umbrella sampling. Free energy calculations were performed using the free energy analysis tools (‘crd_convert’, ‘pathcv_analysis’, ‘mbar_analysis’, and ‘pmf_analysis’) in GENESIS.

Analysis of the transition pathways

The images on the transition from E1P to E2P_dp were grouped into five sub-states (SSs) by using the fixed radius clustering in MMTSB, which classifies the structures using a certain radius

of the Cα RMSD. We confirm that the results are similar with the different radii. Motion Tree (38) was calculated between the neighboring central images using 5.0 Å as the magnitude of the effective nodes. This value was used in the original paper.

The rotational axes and angles between two regions of a protein in Fig 2B were calculated using Domain Selection (39). In this method, the axes and angles of the A- and N-domains from the reference structure are calculated by superimposing on the P-domain.

Data availability

The initial structures of MD simulations, force field parameters, control inputs of MD, TMD, string method, and umbrella sampling, the representative structures of the pathway, and the intermediate structure (image 44) with two Ca^{2+} ions of pathway in GitHub, https://github.com/RikenSugitaLab/SERCA-E1P-E2P-pathway.

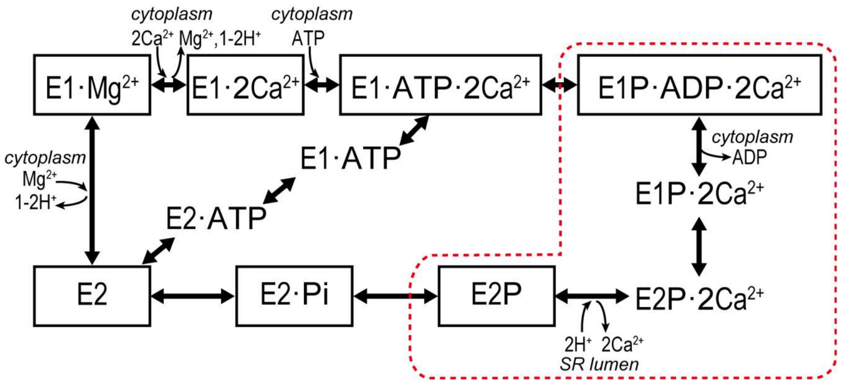

Figure S1. The reaction cycle of sarcoplasmic reticulum Ca^{2+}-ATPase. The physiological states whose crystal structures are available are highlighted in solid boxes. The target chemical steps in the study are surrounded with a dashed red line.

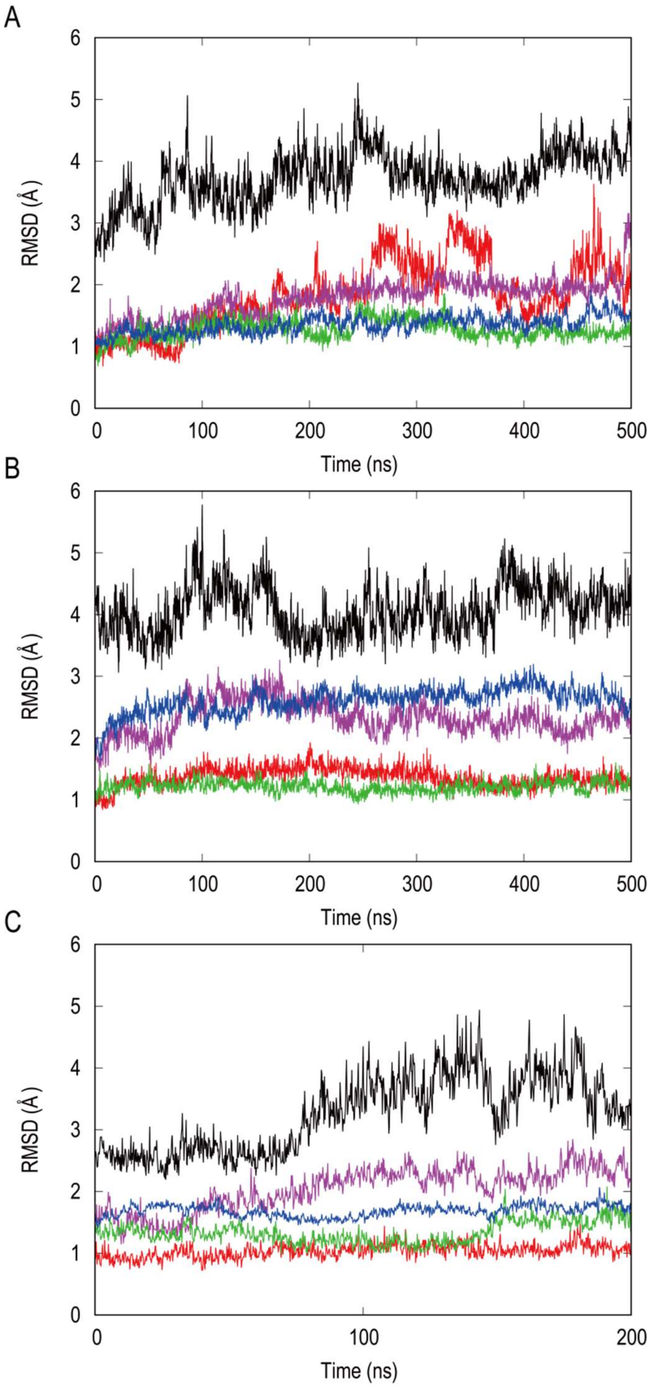

Figure S2. The Cα RMSDs from the starting structures in the simulations of (A) E1P, (B) E2P_dp, and (C) E2P. Black, red, purple, green, and blue lines represent the RMSDs of the whole protein, the A-domain, N-domain, P-domain, and the TM helices, respectively. Flexible loop regions in each domain are removed in the analysis.

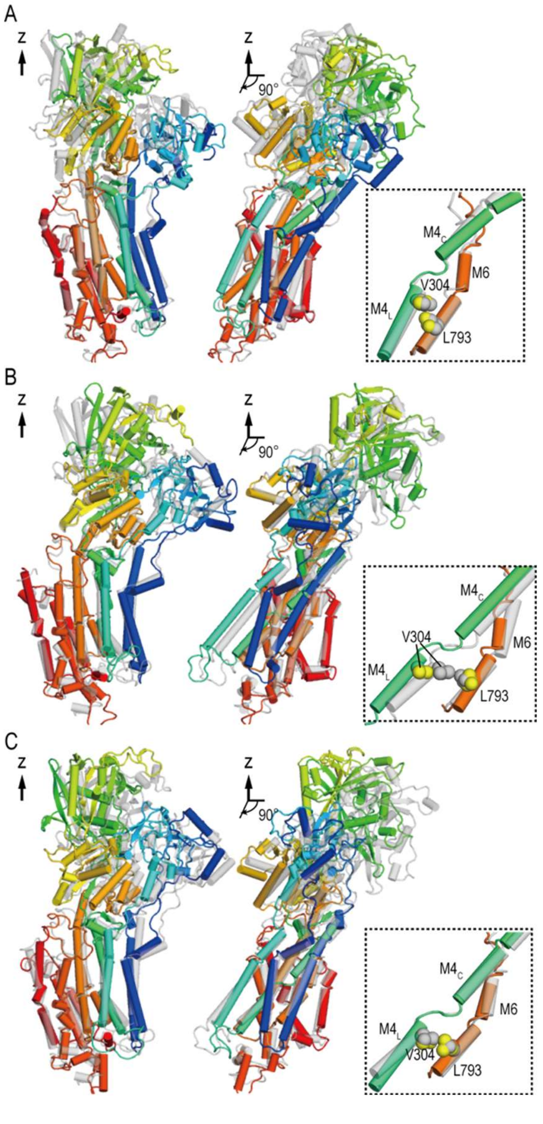

Figure S3. The representative structures in MD simulations of (A) E1P, (B) E2P_dp, and (C) E2P are shown in rainbow. The structures in gray are the starting crystal structures. In dotted boxes, M4 and M6 helices with sidechains Val304 and Leu793 are shown to highlight the luminal gating motions.

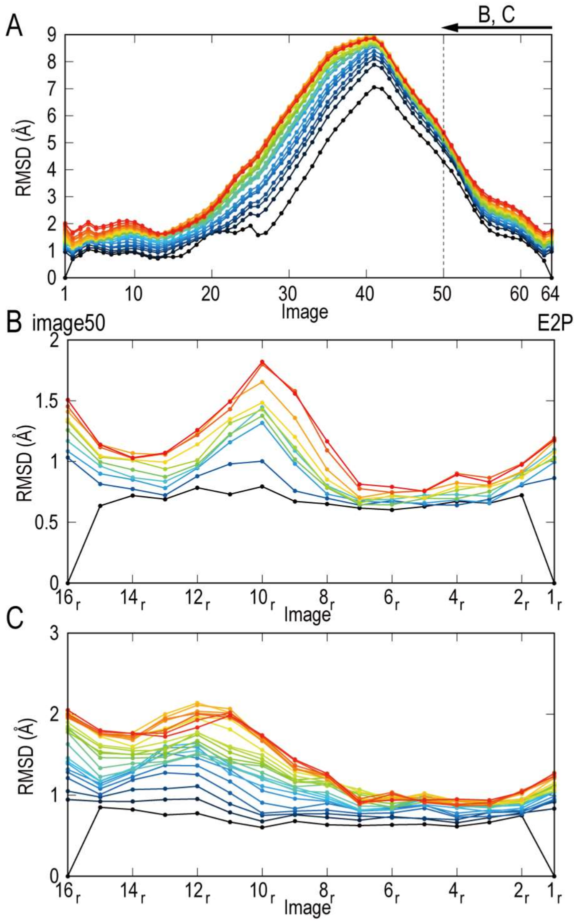

Figure S4. The convergence of the pathways in the mean-force string method calculations (A) from E1P to E2P_dp, (B) from E2P_dp to image 50, and (C) E2P to image 50. The image 50 is taken from the image 50 of the first string-method simulation from E1P to E2P_dp. Line color represents the updates of the string method calculation every 2 ns. Black and red lines represent the pathways for the first 2 ns and the final pathways.

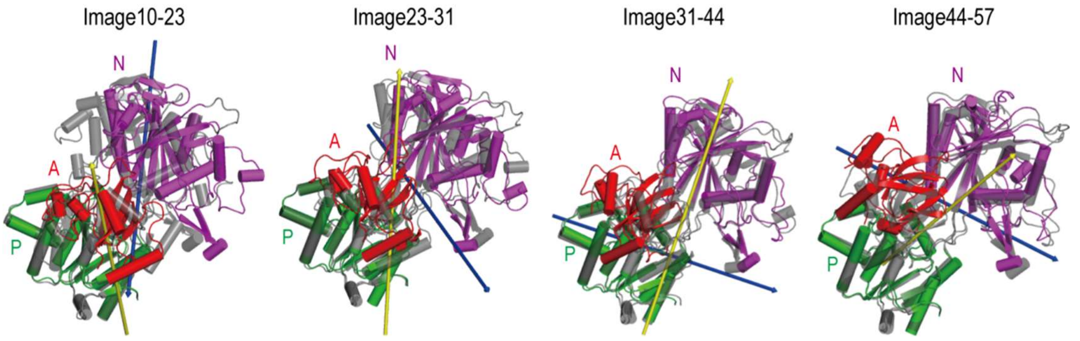

Figure S5. Motions of the cytoplasmic domains between central images in two adjacent SSs at a viewpoint rotated 90˚ from Fig. 2B. Colored and gray structures represent later and earlier states, respectively. Yellow and blue arrows indicate the rotation axes of the A-, and N-domains, respectively, with respect to the P-domain.

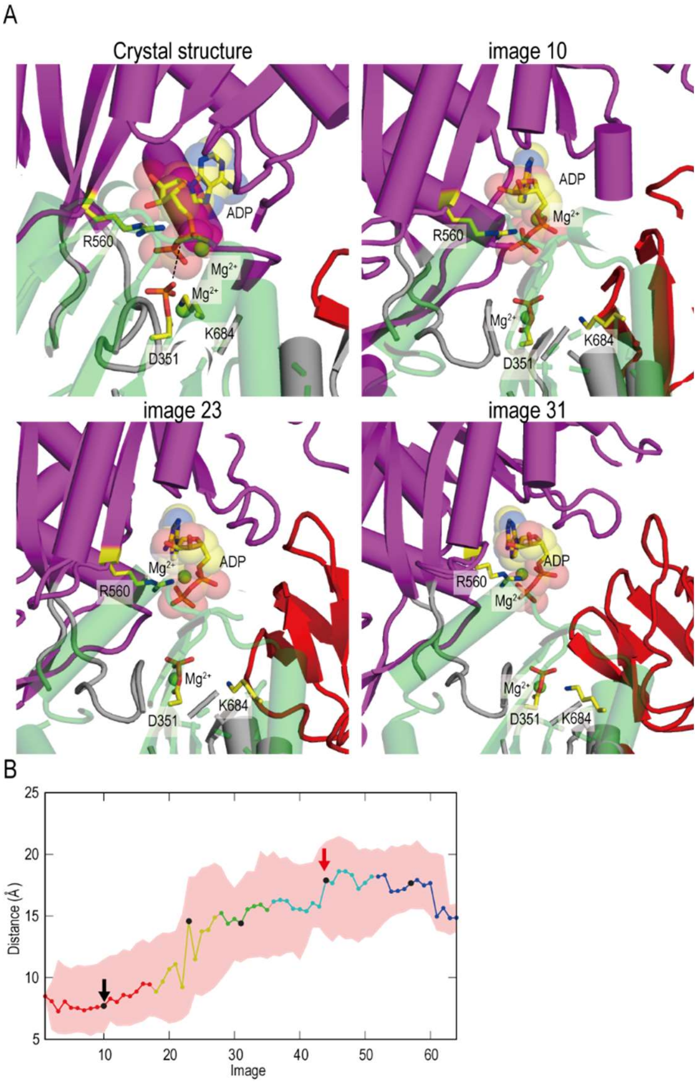

Figure S6. The motion of ADP in the string method calculation from E1P to E2P_dp. (A) The structures of the nucleotide-binding site in the crystal structure of E1P, images 10, 23, and 31 in the string method calculation. (B) The distance between the phosphorus atoms in phosphorylated Asp351 and β-phosphate of ADP. The black and red arrows indicate the beginning and the end of major changes, respectively.

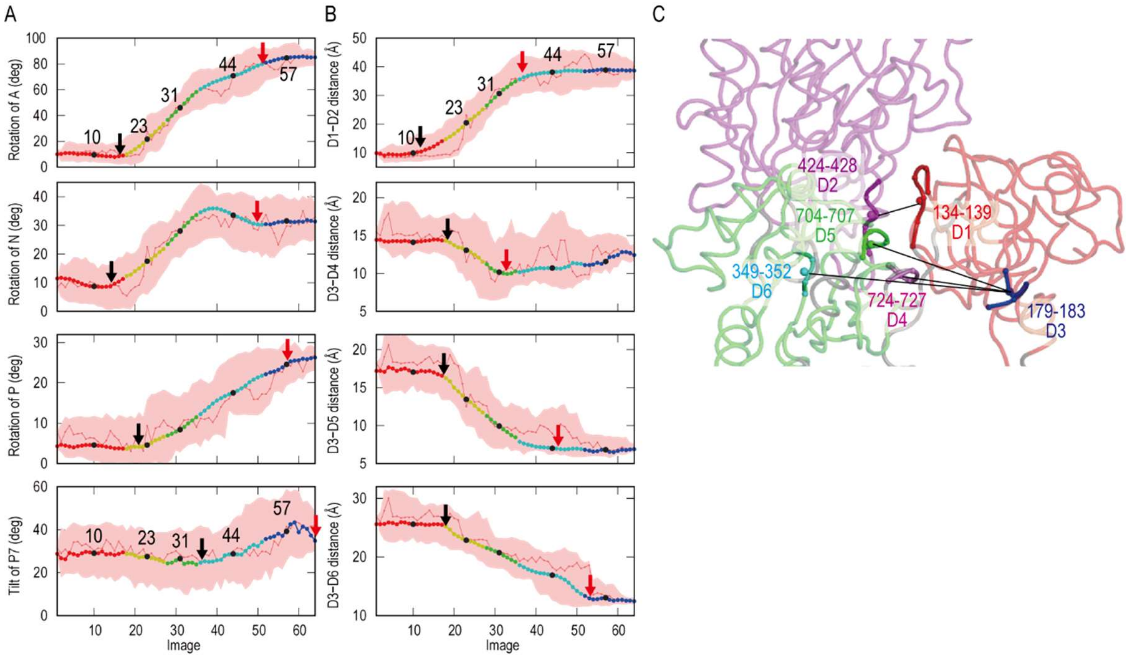

Figure S7. Conformational changes of the cytoplasmic domains in SR Ca^{2+}-ATPase along the transition pathway from E1P to E2P_dp. (A) The rotations of A- (top), N- (2nd top), and P- (3rd top) domains. Each rotation axis of the cytoplasmic domain is calculated by generating a quaternion from structures in the representative MD and an image. Tilt angle of P7 helix (residues 725-731) in P-domain (bottom). The black and red arrows indicate the beginning and the end of major changes, respectively. (B) Distance between geometrical centers of loops in different cytoplasmic domains. (Top) Distance between residues 134-139 in A-domain (D1) and residues 424-428 in N-domain (D2). (2nd top) Distance between residues 179-183 in A-domain (D3) and residues 724-727 in P-domain (D4). (3rd top) Distance between residues 179-183 in A-domain (D3) and residues 704-707 in P-domain (D5). (bottom) Distance between residues 179-183 in A-domain (D3) and residues 349-352 in P-domain (D6). Dots in red, yellow, green, cyan, and blue are images of the five SSs from the converged pathway. Lines in pink and shades represent the average and ranges of sampling in umbrella sampling. The black and red arrows indicate the beginning and the end of major changes, respectively. (C) Definitions of D1-6 for the interdomain distance calculations.

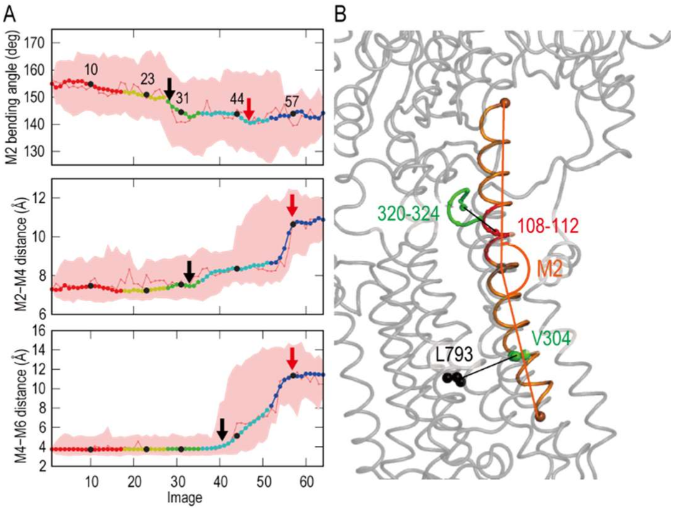

Figure S8. Conformational changes of M2, 4, and 6. (A) Angle defined using the Cα atoms of Val89, Gly105, and Pro124 in M2. (top) Distance between the cytoplasmic parts of M2 and M4. (middle) Minimum distance between sidechain atoms of Val304 in M4 and Leu793 in M6. (bottom) The black and red arrows indicate the beginning and the end of major changes, respectively. (B) Distances and angle in the TM helices are shown in (A).

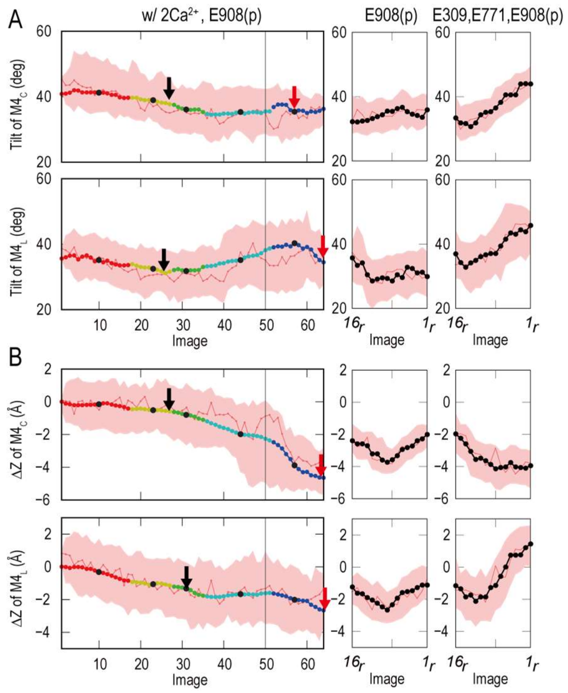

Figure S9. Conformational changes of M4 in the three pathways; (Left) from E1P to E2P_dp, (Middle) from E2P_dp to image 50 (image 16_r), (Right) from E2P to image 50 (image 16_r). (A) Tilt angles of $M4_C$ (residues 309-327, top) and $M4_L$ (residues 293-307, bottom). The black and red arrows indicate the beginning and the end of major changes, respectively. (B) Differences of Z-coordinates of $M4_C$ (residues 309-327, top) and $M4_L$ (residues 293-307, bottom) from the image1. The black and red arrows indicate the beginning and the end of major changes, respectively.

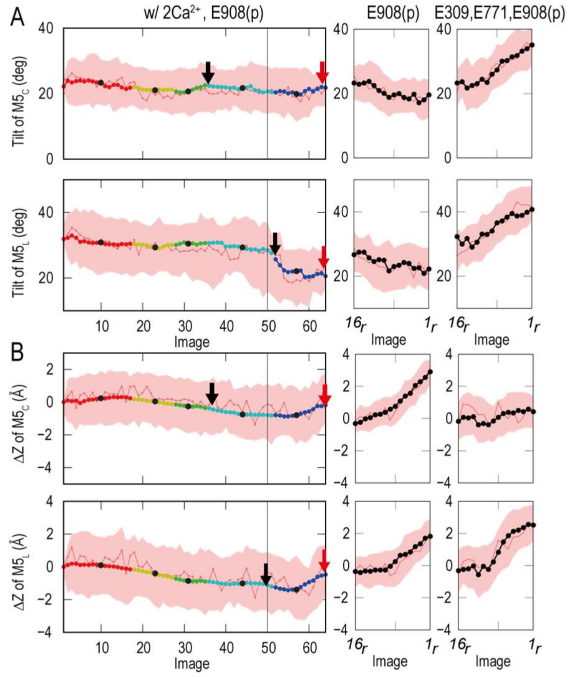

Figure S10. Conformational changes of M5 in the three pathways; (Left) from E1P to E2P_dp, (Middle) from E2P_dp to image 50 (image *16ᵣ*), (Right) from E2P to image 50 (image *16ᵣ*). (A) Tilt angles of $M5_C$ (residues 755-766, top) and $M5_L$ (residues 767-780, bottom). The black and red arrows indicate the beginning and the end of major changes, respectively. (B) Differences of Z-coordinates of $M5_C$ (residues 755-766, top) and $M5_L$ (residues 767-780, bottom) from the image 1. The black and red arrows indicate the beginning and the end of major changes, respectively.

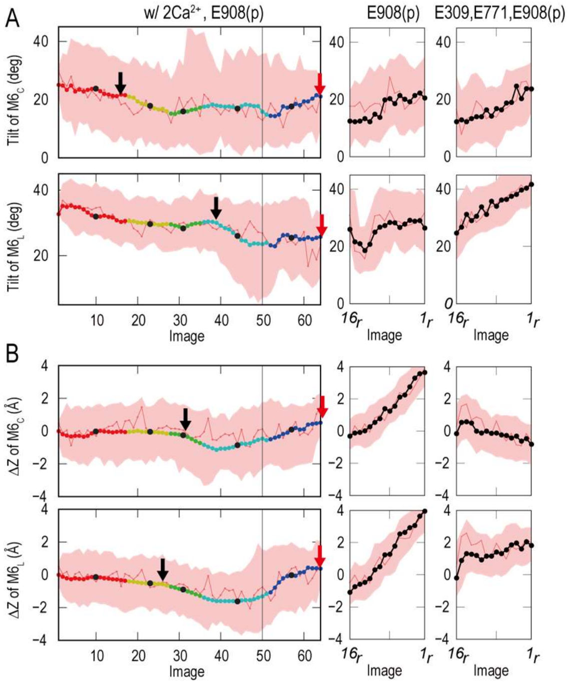

Figure S11. Conformational changes of M6 in the three pathways; (Left) from E1P to E2P_dp, (Middle) from E2P_dp to image 50 (image *16_r*), (Right) from E2P to image 50 (image *16_r*). (A) Tilt angles of $M6_C$ (residues 802-807, top) and $M6_L$ (residues 790-799, bottom). The black and red arrows indicate the beginning and the end of major changes, respectively. (B) Differences of Z-coordinates of $M6_C$ (residues 802-807, top) and $M6_L$ (residues 790-799, bottom) from the image 1. The black and red arrows indicate the beginning and the end of major changes, respectively.

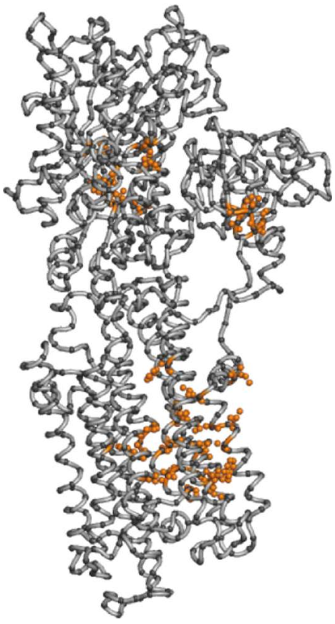

Figure S12. The Cα atoms and the carbon atoms of sidechains that are used to define the collective variables (CVs) in the mean-force string method and umbrella sampling method are shown in grey and orange spheres, respectively.

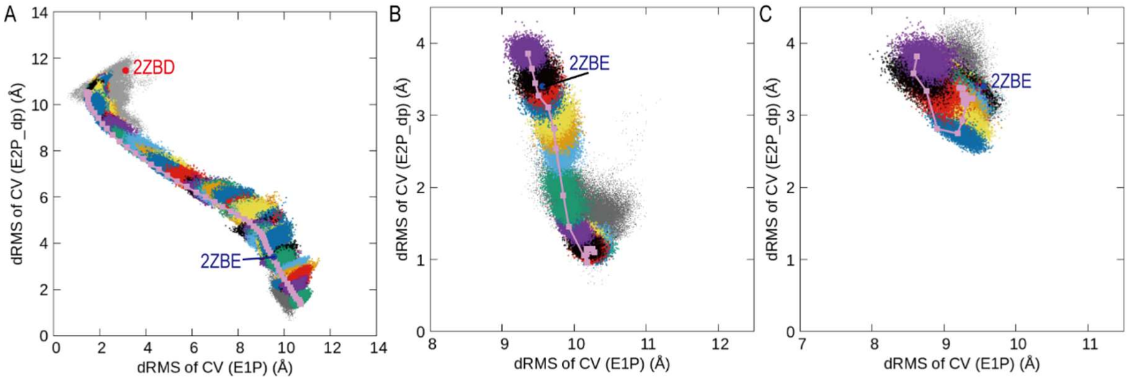

Figure S13. Conformational sampling spaces of the mean-force string method and umbrella sampling for the transition paths (A) from E1P to E2P_dp, (B) from E2P_dp to image 50, and (C) from E2P to image 50. The optimized pathways from the string method are depicted with a pink line, whereas each snapshot of the umbrella sampling in each window (image) is shown as a dot with different color. The two crystal structures (2ZBD and 2ZBE) are shown as dots in red and blue, respectively. The two axes are defined as *dRMS* (distance root mean square displacement) from the representative structures in MD simulations of E1P and E2P_dp.

Table S1. Simulation systems of SR Ca^{2+}-ATPase in E1P·ADP·2Ca^{2+} and E2P.

System	PDB ID	Protonated residues	Ca^{2+}-binding sites	Nucleotide-binding site	# of atoms
E1P	2ZBD	E908	2Ca^{2+}	ADP, 2Mg^{2+}	297,475
E2P_dp	2ZBE	E908	N/A	Mg^{2+}	297,523
E2P	2ZBE	E309, E771, E908	N/A	Mg^{2+}	297,529

Table S2. Summary of the RMSDs between PDB structures and representative structures in MD simulations. Protonated residues in individual simulations are described in Table S1. The RMSD from the crystal structure is computed using all the Cα atoms of the representative structure of each simulation. The RMSD in TMD simulation is computed for the final snapshot.

Method	Protonation state	Starting (and target) structure(s)	Simulation Length (ns)	RMSD (Å) from 2ZBD	RMSD (Å) from 2ZBE
PDB	-	2ZBD	-	0.00	10.61
PDB	-	2ZBE	-	10.61	0.00
MD	E1P	E1P	500	4.09	8.94
MD	E2P_dp	E2P_dp	500	10.92	4.62
MD	E2P	E2P	200	9.34	4.01
TMD	E1P	E1P → E2P_dp	200	10.92	4.63
TMD	E2P_dp	E2P_dp → image 50*	20	10.92	4.96
TMD	E2P	E2P→ image 50*	20	10.92	4.97

(*) The image 50 represents a representative structure taken from the image 50 of the first MD simulation with mean-force string method.

Table S3. Summary of MD simulations based on the string method and umbrella sampling.

Method		(1) E1P → E2P_dp	(2) E2P_dp → image 50*	(3) E2P → image 50*
String method	# of images	64	16	16
	Equilibration (ns/image)	40	30	30
	Path optimization (ns/image)	60	20	50
	Total (ns)	6,400	800	1,280
Umbrella sampling	# of images	64	16	16
	Equilibration (ns/image)	4	4	4
	Free energy calc. (ns/image)	46	46	46
	Force constant (kcal/mol/Å^2)	0.002	0.02	0.005
	Total (ns)	3,200	800	800

(*) The image 50 represents a representative structure taken from the image 50 of the first MD simulation with mean-force string method.

SI References

1. C. Toyoshima, H. Nomura, T. Tsuda, Lumenal gating mechanism revealed in calcium pump crystal structures with phosphate analogues. *Nature* **432**, 361-368 (2004).
2. C. Toyoshima, Y. Norimatsu, S. Iwasawa, T. Tsuda, H. Ogawa, How processing of aspartylphosphate is coupled to lumenal gating of the ion pathway in the calcium pump. *Proceedings of the National Academy of Sciences of the United States of America* **104**, 19831-19836 (2007).
3. K. Yamasaki, G. L. Wang, T. Daiho, S. Danko, H. Suzuki, Roles of Tyr(122)-hydrophobic Cluster and K^+ Binding in Ca^{2+}-releasing Process of ADP-insensitive Phosphoenzyme of Sarcoplasmic Reticulum Ca^{2+}-ATPase. *Journal of Biological Chemistry* **283**, 29144-29155 (2008).
4. M. A. Lomize, I. D. Pogozheva, H. Joo, H. I. Mosberg, A. L. Lomize, OPM database and PPM web server: resources for positioning of proteins in membranes. *Nucleic Acids Research* **40**, D370-D376 (2012).
5. Y. Sugita, N. Miyashita, M. Ikeguchi, A. Kidera, C. Toyoshima, Protonation of the acidic residues in the transmembrane cation-binding sites of the Ca^{2+} pump. *Journal of the American Chemical Society* **127**, 6150-6151 (2005).
6. C. R. Sondergaard, M. H. M. Olsson, M. Rostkowski, J. H. Jensen, Improved Treatment of Ligands and Coupling Effects in Empirical Calculation and Rationalization of pK(a) Values. *Journal of Chemical Theory and Computation* **7**, 2284-2295 (2011).
7. M. H. M. Olsson, C. R. Sondergaard, M. Rostkowski, J. H. Jensen, PROPKA3: Consistent Treatment of Internal and Surface Residues in Empirical pK(a) Predictions. *Journal of Chemical Theory and Computation* **7**, 525-537 (2011).
8. X. Yu, L. N. Hao, G. Inesi, A Pk Change of Acidic Residues Contributes to Cation Countertransport in the Ca-Atpase of Sarcoplasmic-Reticulum - Role of H^+ in Ca^{2+}-Atpase Countertransport. *Journal of Biological Chemistry* **269**, 16656-16661 (1994).
9. G. Inesi, F. Tadini-Buoninsegni, Ca^{2+}/H^+ exchange, lumenal Ca^{2+} release and Ca^{2+}/ATP coupling ratios in the sarcoplasmic reticulum ATPase. *Journal of Cell Communication and Signaling* **8**, 5-11 (2014).
10. L. Zhang, J. Hermans, Hydrophilicity of cavities in proteins. *Proteins-Structure Function and Genetics* **24**, 433-438 (1996).
11. J. Jung, T. Mori, C. Kobayashi, Y. Matsunaga, T. Yoda, M. Feig, Y. Sugita, GENESIS: a hybrid-parallel and multi-scale molecular dynamics simulator with enhanced sampling algorithms for biomolecular and cellular simulations. *Wiley Interdisciplinary Reviews: Computational Molecular Science* **5**, 310-323 (2015).
12. J. Jung, A. Naruse, C. Kobayashi, Y. Sugita, Graphics Processing Unit Acceleration and Parallelization of GENESIS for Large-Scale Molecular Dynamics Simulations. *Journal of Chemical Theory and Computation* **12**, 4947-4958 (2016).
13. C. Kobayashi, J. Jung, Y. Matsunaga, T. Mori, T. Ando, K. Tamura, M. Kamiya, Y. Sugita, GENESIS 1.1: A hybrid-parallel molecular dynamics simulator with enhanced sampling algorithms on multiple computational platforms. *Journal of Computational Chemistry* **38**, 2193-2206 (2017).
14. J. B. Klauda, R. M. Venable, J. A. Freites, J. W. O'Connor, D. J. Tobias, C. Mondragon-Ramirez, I. Vorobyov, A. D. MacKerell, R. W. Pastor, Update of the CHARMM All-Atom

Additive Force Field for Lipids: Validation on Six Lipid Types. *Journal of Physical Chemistry B* **114**, 7830-7843 (2010).

15. R. B. Best, X. Zhu, J. Shim, P. E. M. Lopes, J. Mittal, M. Feig, A. D. MacKerell, Optimization of the Additive CHARMM All-Atom Protein Force Field Targeting Improved Sampling of the Backbone ϕ, φ and Side-Chain χ_1 and χ_2 Dihedral Angles. *Journal of Chemical Theory and Computation* **8**, 3257-3273 (2012).
16. D. J. Price, C. L. Brooks, A modified TIP3P water potential for simulation with Ewald summation. *Journal of Chemical Physics* **121**, 10096-10103 (2004).
17. Y. Komuro, S. Re, C. Kobayashi, E. Muneyuki, Y. Sugita, CHARMM Force-Fields with Modified Polyphosphate Parameters Allow Stable Simulation of the ATP-Bound Structure of Ca^{2+}-ATPase. *Journal of Chemical Theory and Computation* **10**, 4133-4142 (2014).
18. P. Li, B. P. Roberts, D. K. Chakravorty, K. M. Merz, Rational Design of Particle Mesh Ewald Compatible Lennard-Jones Parameters for +2 Metal Cations in Explicit Solvent. *Journal of Chemical Theory and Computation* **9**, 2733-2748 (2013).
19. J. P. Ryckaert, G. Ciccotti, H. J. C. Berendsen, Numerical-Integration of Cartesian Equations of Motion of a System with Constraints - Molecular-Dynamics of N-Alkanes. *Journal of Computational Physics* **23**, 327-341 (1977).
20. H. C. Andersen, Rattle - a Velocity Version of the Shake Algorithm for Molecular-Dynamics Calculations. *Journal of Computational Physics* **52**, 24-34 (1983).
21. S. Miyamoto, P. A. Kollman, Settle - an Analytical Version of the Shake and Rattle Algorithm for Rigid Water Models. *Journal of Computational Chemistry* **13**, 952-962 (1992).
22. T. Darden, D. York, L. Pedersen, Particle Mesh Ewald - an N.Log(N) Method for Ewald Sums in Large Systems. *Journal of Chemical Physics* **98**, 10089-10092 (1993).
23. U. Essmann, L. Perera, M. L. Berkowitz, T. Darden, H. Lee, L. G. Pedersen, A Smooth Particle Mesh Ewald Method. *Journal of Chemical Physics* **103**, 8577-8593 (1995).
24. P. J. Steinbach, B. R. Brooks, New Spherical-Cutoff Methods for Long-Range Forces in Macromolecular Simulation. *Journal of Computational Chemistry* **15**, 667-683 (1994).
25. M. Tuckerman, B. J. Berne, G. J. Martyna, Reversible Multiple Time Scale Molecular-Dynamics. *Journal of Chemical Physics* **97**, 1990-2001 (1992).
26. J. Jung, C. Kobayashi, Y. Sugita, Kinetic energy definition in velocity Verlet integration for accurate pressure evaluation. *Journal of Chemical Physics* **148** (2018).
27. D. Quigley, M. I. J. Probert, Langevin dynamics in constant pressure extended systems. *Journal of Chemical Physics* **120**, 11432-11441 (2004).
28. G. Bussi, D. Donadio, M. Parrinello, Canonical sampling through velocity rescaling. *Journal of Chemical Physics* **126**, 014101 (2007).
29. M. Feig, J. Karanicolas, C. L. Brooks (2001) MMTSB Tool Set (The Scripps Research Institute).
30. M. Feig, J. Karanicolas, C. L. Brooks, MMTSB Tool Set: enhanced sampling and multiscale modeling methods for applications in structural biology. *Journal of Molecular Graphics & Modelling* **22**, 377-395 (2004).
31. L. Maragliano, A. Fischer, E. Vanden-Eijnden, G. Ciccotti, String method in collective variables: Minimum free energy paths and isocommittor surfaces. *Journal of Chemical Physics* **125** (2006).

32. G. M. Torrie, J. P. Valleau, Non-Physical Sampling Distributions in Monte-Carlo Free-Energy Estimation - Umbrella Sampling. *Journal of Computational Physics* **23**, 187-199 (1977).
33. J. Schlitter, M. Engels, P. Kruger, E. Jacoby, A. Wollmer, Targeted Molecular-Dynamics Simulation of Conformational Change - Application to the T[--]R Transition in Insulin. *Molecular Simulation* **10**, 291-& (1993).
34. C. Kobayashi, R. Koike, M. Ota, Y. Sugita, Hierarchical domain-motion analysis of conformational changes in sarcoplasmic reticulum Ca^{2+}-ATPase. *Proteins-Structure Function and Bioinformatics* **83**, 746-756 (2015).
35. S. Park, T. Kim, W. Im, Transmembrane Helix Assembly by Window Exchange Umbrella Sampling. *Physical Review Letters* **108** (2012).
36. M. R. Shirts, J. D. Chodera, Statistically optimal analysis of samples from multiple equilibrium states. *Journal of Chemical Physics* **129** (2008).
37. Y. Matsunaga, H. Fujisaki, T. Terada, T. Furuta, K. Moritsugu, A. Kidera, Minimum Free Energy Path of Ligand-Induced Transition in Adenylate Kinase. *Plos Computational Biology* **8** (2012).
38. R. Koike, M. Ota, A. Kidera, Hierarchical description and extensive classification of protein structural changes by motion tree. *Journal of Molecular Biology* **426**, 752-762 (2014).
39. S. Hayward, A. Kitao, H. J. C. Berendsen, Model-free methods of analyzing domain motions in proteins from simulation: A comparison of normal mode analysis and molecular dynamics simulation of lysozyme. *Proteins-Structure Function and Genetics* **27**, 425-437 (1997).
